# Nutritional systems biology of type 2 diabetes

**DOI:** 10.1007/s12263-015-0481-3

**Published:** 2015-07-24

**Authors:** Yuqi Zhao, Rio Elizabeth Barrere-Cain, Xia Yang

**Affiliations:** Department of Integrative Biology and Physiology, University of California, Los Angeles, Los Angeles, CA 90095 USA

**Keywords:** Nutrition, Diet, Systems biology, Type 2 diabetes, Omics

## Abstract

Type 2 diabetes (T2D) has become an increasingly challenging health burden due to its high morbidity, mortality, and heightened prevalence worldwide. Although dietary and nutritional imbalances have long been recognized as key risk factors for T2D, the underlying mechanisms remain unclear. The advent of nutritional systems biology, a field that aims to elucidate the interactions between dietary nutrients and endogenous molecular entities in disease-related tissues, offers unique opportunities to unravel the complex mechanisms underlying the health-modifying capacities of nutritional molecules. The recent revolutionary advances in omics technologies have particularly empowered this incipient field. In this review, we discuss the applications of multi-omics approaches toward a systems-level understanding of how dietary patterns and particular nutrients modulate the risk of T2D. We focus on nutritional studies utilizing transcriptomics, epigenomomics, proteomics, metabolomics, and microbiomics, and integration of diverse omics technologies. We also summarize the potential molecular mechanisms through which nutritional imbalances contribute to T2D pathogenesis based on these studies. Finally, we discuss the remaining challenges of nutritional systems biology and how the field can be optimized to further our understanding of T2D and guide disease management via nutritional interventions.

## Introduction

Type 2 diabetes (T2D), defined as hyperglycemia resulting from compromised insulin utilization (insulin resistance, IR) coupled with insufficient compensatory insulin production, is the common form of diabetes mellitus. It is one of the most pressing public health challenges we are facing worldwide. According to the World Health Organization (WHO; www.who.int) and the Center for Disease Control and Prevention (CDC; www.cdc.gov), T2D is among the top ten leading causes of death in the world and in the USA. Recent estimates by the International Diabetes Federation (http://www.idf.org) indicate that in 2013, 382 million adults aged 20–70 years had T2D, and by the year 2030, the number is expected to reach 438 million. More alarmingly, prediabetes is increasingly prevalent among children, adolescents, and younger adults (Ardisson Korat et al. 2014). The long-term consequences and comorbidities of T2D include retinopathy, nephropathy, neuropathy, hypertension, dyslipidemia, cerebrovascular disease, cardiovascular disease, and peripheral vascular disease (Alberti and Zimmet 2013; Atkins et al. 2010; Donath and Shoelson 2011).

Epidemiological studies have shown that nearly 90 % of T2D cases can be attributed to five major lifestyle factors: diet, physical activity, smoking, overweight or obesity, and alcohol consumption (Chen et al. 2012a; Hu 2011). Among these, diet is particularly important given that T2D is a disease rooted in dysfunctional metabolism and utilization of energy fuel, and given that dietary imbalance in both quantity and quality is also an established risk factor for overweight or obesity which is tightly linked to T2D. Both epidemiological studies and clinical trials in T2D patients indicate that insulin sensitivity or other glycemic traits are strongly affected by dietary patterns. In particular, T2D is more prevalent in populations consuming the so-called Western or conservative diet, which is high in carbohydrates (from refined grains and sugar), red meat, and saturated fat. On the other hand, dietary interventions involving increased polyunsaturated fat and fiber reduce T2D risk (Hu 2011; Kastorini and Panagiotakos 2009). Despite the ample epidemiological and clinical evidence supporting the diet–disease association, exactly how dietary patterns alter molecular processes that are responsible for glucose homeostasis and insulin function and ultimately lead to T2D remains unclear. A better understanding of these molecular events can provide fundamental clues about T2D pathogenesis and help uncover novel therapeutic targets. In addition, fully understanding the molecular impact of diverse types of dietary patterns and nutrients may facilitate the development of nutritional remedies for T2D as well as preventative strategies for curbing prediabetes and T2D epidemic.

However, exploring the molecular-level associations between nutrients and T2D can be difficult. First, most dietary nutrients are complex products and consist of a mixture of different components, making their effects far more complicated and unpredictable than the direct effects from single molecules. Second, as dietary nutrients provide the most fundamental building blocks and fuels for the body and participate in diverse physiological functions, their broad and complex impact on and interactions with the genome (DNA elements), epigenome (modifications of DNA elements), proteome (protein products of coding genes), metabolome (metabolite products of metabolic pathways), and even the microbiome (bacteria species interacting with host) in multiple types of cells, tissues, and organ systems are expected. Only genome-wide approaches that have the capacity to capture these multi-dimensional signals can help achieve a systems-level understanding of the molecular underpinnings of diet-induced T2D.

**Fig. 1:**
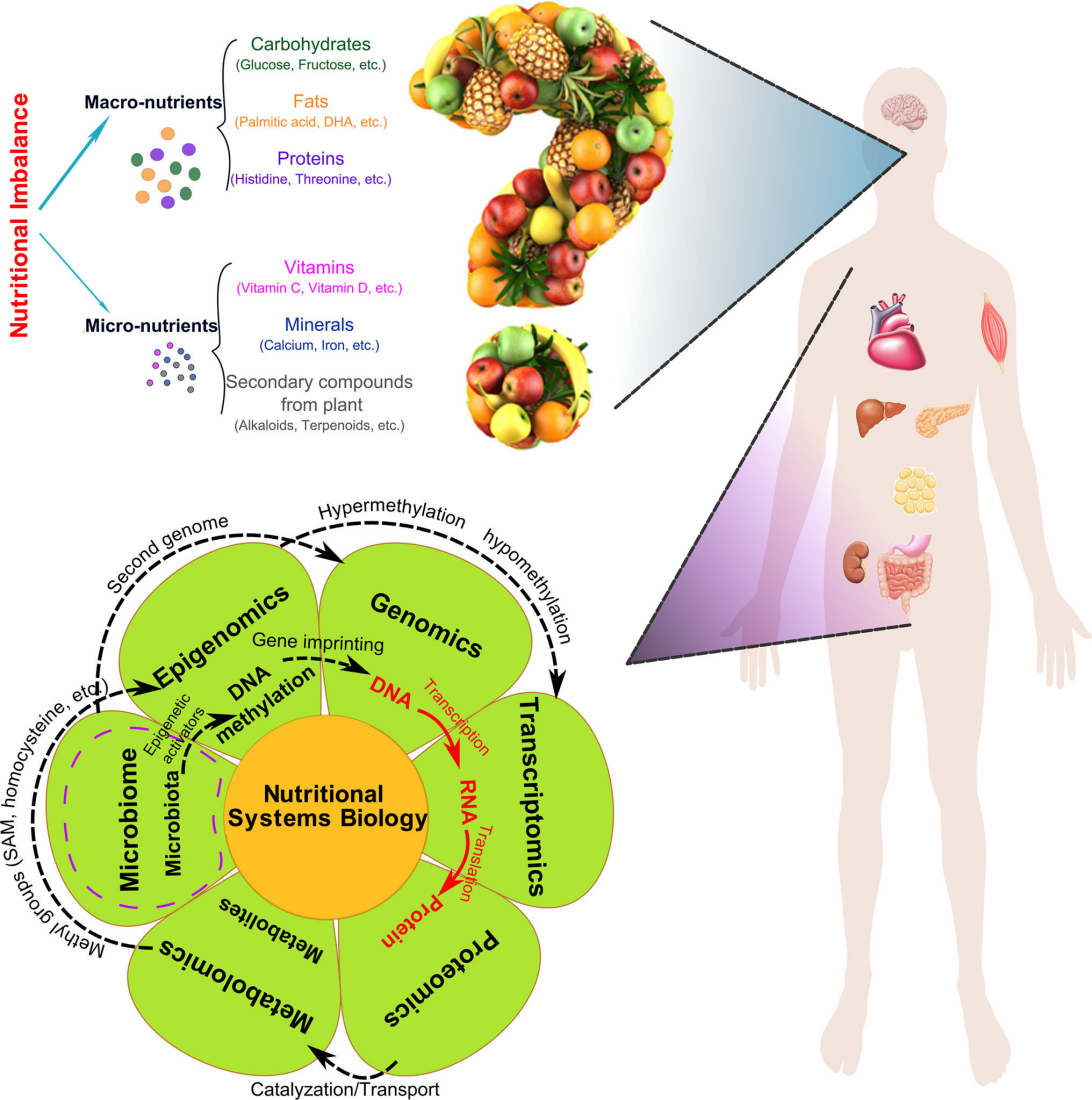
Nutritional factors and omics technologies used in nutritional systems biology

Recently, nutritional systems biology has been introduced to help address the challenges of nutritional research outlined above (Panagiotou and Nielsen 2009). In essence, nutritional systems biology aims to assess nutritional intake and then measure the consequences as accurate transcriptomic, epigenomic, proteomic, and metabolomic signals (Fig. 1). These signals can be integrated into comprehensive, tissue-specific network views to depict the molecular mechanistic maps of nutritional variations. The arrival of this concept is timely given the maturation of diverse omics technologies over the past decade.

In this review, we summarize recent progresses in the applications of nutritional systems biology in T2D research. Because the majority of the existing nutritional studies focus on a single level of omics data, we focus on nutritranscriptomics, nutriproteomics, nutrimetabolomics, nutriepigenomics, and nutrimicrobiomics studies that involve genome-wide scans of transcriptome, proteome, metabolome, epigenome, and microbiome in response to dietary modulation, respectively. Of note, although nutrigenetics that investigates interactions between nutrients and genetic variation (i.e., how DNA changes determine differential responses to nutrition) is also an important part of nutritional systems biology, we do not cover this topic here due to the lack of genome-wide investigations at present and the low reproducibility of candidate gene-based studies (Ioannidis et al. 2011). We first delineate the intrinsic relationships between nutrients and different levels of omics and then discuss the underlying concepts, technologies, and example studies at each nutri-omics level. Subsequently, we summarize endeavors in higher-level systems analyses that harmonize multi-omics datasets to derive more comprehensive views of interactions among molecular elements in response to nutritional modulations. Finally, we highlight the remaining challenges and future directions for nutritional systems biology.

## Relationships between different categories of omics

Although it may appear to be a daunting task to piece together the molecular signatures of dietary patterns or nutrients given the large amount of information across multiple levels of molecular entities, the intrinsic relationships between molecular entities can facilitate data modeling and interpretation (Fig. 1).

According to the central dogma, DNA encodes and is transcribed into mRNA, which subsequently encodes and translates into proteins. Accumulating evidence in the past few decades has led to modifications to the linear model of the central dogma. For example, the epigenome, considered the second dimension to our genome and consisting of multiple sequence-independent processes that regulate gene expression, does not alter the coding information of DNA sequences but contributes to transcriptional regulation through chemical modifications to the DNA and histone proteins (Rivera and Ren 2013) or through noncoding RNAs such as microRNAs and long noncoding RNAs (lncRNAs) (Cech and Steitz 2014). These findings challenge the belief that both necessary and sufficient information for cellular function is contained in the gene sequence.

As the end products of gene regulation, proteins are the basic functional units of cellular processes and the primary players in structural, biochemical, and signaling functions. For example, all transcriptional and translational processes involved in the central dogma and epigenomic mechanisms described above require essential protein products such as transcription factors, DNA methyltransferases, histone deacetylases, and DNA/RNA binding proteins. Additionally, many proteins involved in metabolic reactions possess enzymatic activities that metabolize nutrients into various metabolites. The gut microbiome, considered as our second genome, also produces enzymes that assist in metabolism and influence metabolite diversity and quantity. Metabolites, in turn, play important roles in gene expression regulation by contributing to modifications of the epigenome that subsequently change the transcriptional regulatory machinery.

Although each omics technology only provides a snapshot of signatures of the same molecular category (e.g., differentially expressed genes) for a given diet or a particular nutrient, a comprehensive view can be obtained using the between-category interactions. For instance, known pair-wise relations, such as genetics to gene expression, epigenetics to gene expression, genetics to protein, gene expression to protein, protein to metabolite, and metabolite to epigenome, can be examined and modeled based on the biological information flow. When all types of molecular data are gathered, it is possible to model the data simultaneously based on both the data patterns (e.g., correlative relations between genes) and biological relationship (e.g., a protein is known to be responsible for the production of a metabolite).

To date, the majority of the nutritional studies focus on a single omics data type. Therefore, in the subsequent sections, we first summarize the separate applications of nutritranscriptomic, nutriepigenomic, nutriproteomic, nutrimetabolomic, and nutrimicrobiotic studies in T2D and then review the multi-omics systems-level studies that are currently available.

## Nutritranscriptomics in T2D

Transcriptomics, that is, the simultaneous measurement of nearly all genes expressed in a given cell, tissue, or organism, has been the most successful technology applied to nutritional systems biology in the recent decades (Capozzi and Bordoni 2013). Transcriptomics covers the step of passing information from DNA to RNA. DNA microarrays and, more recently, high-throughput RNA sequencing (RNA-Seq) technologies are the most commonly used transcriptomics tools in nutritional studies. Microarrays are capable of measuring the expression levels of thousands of genes simultaneously by hybridizing total mRNAs from biological samples to predesigned gene-specific probes. More recently, sequencing-based RNA-Seq has become a revolutionary approach to transcriptome profiling because it is more sensitive and has a broader dynamic range than microarray tools (Ozsolak and Milos 2011; Wang et al. 2009). More importantly, RNA-Seq can detect gene transcript signals from previously unannotated genes and also allow analysis of transcripts from either the forward or the reverse strand, offering higher discovery potential compared to microarrays which focus on previously known genes and transcripts with prespecified directionality. To date, the most commonly used RNA-seq technologies such as the Illumina platform typically generate short-reads in the range of a few hundred base pairs and impose challenges in the precise reconstruction of transcript structures. Long-read RNA-seq technologies such as Pacific Biosciences’ single-molecule real-time sequencing are capable of generating reads of more than 20,000 base pairs and can facilitate de novo assembly (Eid et al. 2009; Tilgner et al. 2014). Here we summarize key applications of transcriptomics in nutritional studies primarily in the last 4 years that are relevant to T2D research. The findings from nutritranscriptomic studies of high-fat diet (HFD) and high-sugar diets, which have been under more intense investigations, are summarized in Table 1.

**Table 1:** Differentially expressed biological processes and key genes in response to diet imbalance

Tissue	High-fat diet	High-sucrose/high-fructose diet
Liver	Glycolysis, Krebs cycle, β oxidation, fatty acid metabolism, cholesterol biosynthesis, oxidative phosphorylation, insulin signaling, glucose regulation, lipid metabolism, adipogenesis, PPAR signaling, bile acid metabolism, steroid hormone metabolism, proteasome, the ubiquitin-mediated proteolysis, peroxisome, metabolism amino acids, cytokine receptor interactions, cell differentiation, immune response, inflammatory pathways (Chang et al. 2014; de Fourmestraux et al. 2004; Inoue et al. 2005; Kim et al. 2004; Lee et al. 2012; Matsui et al. 2005; Miller et al. 2013; Nojima et al. 2013a; Patsouris et al. 2006; Waller-Evans et al. 2013a, b; Xia et al. 2014)	*Sucrose*: Lipid metabolism, amino acid metabolism, steroid metabolism, transcription, cell cycle, apoptosis, signal transduction, redox control, immune response (Nojima et al. 2013b) *Fructose*: Lipid metabolism (fatty acid metabolism, biosynthesis of steroids, synthesis and degradation of ketone bodies, FA elongation in mitochondria, bile acid synthesis), cell cycle regulation (Oster et al. 2012a, b)
Adipose	Inflammatory response, response to external stimulus, immune system, lipid metabolism, fatty acid synthesis and transport, triglyceride cycling, TCA cycle, PPAR signaling, leukocyte activation, toll-like receptor signaling, cytokine–cytokine receptor interaction, mitochondrial biogenesis, cell differentiation (Ding et al. 2013; Koza et al. 2006; Lee et al. 2012; Matsui et al. 2005; Morine et al. 2013)	–
Muscle	Glycolysis, Krebs cycle, β oxidation, fatty acid synthesis and oxidation, mitochondrial oxidative phosphorylation, mitochondrial biogenesis, Cytokine signaling, inflammatory response, protein metabolism and modification, nucleic acid metabolism, starch and sucrose metabolism, phosphorylation of insulin signaling protein kinase B, cell differentiation (de Fourmestraux et al. 2004; de Wilde et al. 2009; Latouche et al. 2014; Lee et al. 2012; Oh and Yun 2012; Sparks et al. 2005)	–
Gastrointestinal tract	Immunity, lipid and fatty acid metabolism, signal transduction, olfaction (Cui et al. 2009; Primeaux et al. 2013)	–
Islet/pancreas	Cell cycle/growth/proliferation, inflammation/immune response, ER stress, extracellular matrix (Barbosa-Sampaio et al. 2013; Roat et al. 2014; Sims et al. 2013)	*Sucrose*: Apoptosis, cell cycle, glycolysis, Krebs cycle, and lipid metabolism (Wolden-Kirk et al. 2014)
Hypothalamus	Transcription, neuropeptide signaling, cell adhesion, glucose homeostasis, regulation of glucose sensitivity and transport, corticotrophin releasing hormone (Dearden and Balthasar 2014; Lee et al. 2010)	–

The transition from a “lean, healthy” diet low in fat and carbohydrates to HFD has been robustly linked to many common, complex diseases or pathological conditions and thus has been the focus of nutritranscriptomic studies in the context of obesity, insulin resistance (IR), and diabetes (Wen et al. 2011). Numerous studies have investigated the HFD-induced gene expression changes in various T2D-related tissues including liver, adipose, muscle, islet, and hypothalamus. Due to differences in the length of intervention, the amount of fat in the diets used, the individual tissues examined, and the animal models involved, there is variability in the top differentially expressed genes detected between studies. However, certain consistent genes have been observed in at least two studies, including *PCK1* (phosphoenolpyruvate carboxykinase 1), *COL1A1* (collagen, type I, alpha 1), and *PPARG*. In addition to these individual genes, multiple perturbed biological pathways including lipid metabolism, inflammatory processes, and cell cycle regulation have been robustly detected by multiple studies. Moreover, tissue-specific patterns have emerged. For example, inflammatory and immune processes are captured more frequently in the adipose tissue, while lipid metabolism, oxidative phosphorylation, peroxisome proliferator-activating receptor (PPAR) signaling, and insulin signaling appear to be more affected in liver. Regarding the potential mechanisms underlying the effect of HFD on inflammation in adipose tissue, Sun et al. (2012) thoroughly reviewed this topic and summarized that HFD-induced lipid overload may initiate inflammation via its diverse effects on inflammasomes, innate receptors, nuclear receptors, cell death, ER stress, and gut microbiota. Furthermore, transcriptomic response to HFD exhibits genetic background- or strain-specific patterns. For instance, the liver transcriptome of C57BL/6J (sensitive to HFD-induced IR) and that of BALB/c (resistant to HFD-induced IR) showed opposite expression patterns in genes involved in proteasome and ubiquitin-mediated proteolysis pathways (Waller-Evans et al. 2013a, b). A study of islet transcriptome also revealed striking differences between C57BL/6J (IR sensitive) and BLKS (resistant to HFD-induced IR) in pathways related to cell cycle, growth, proliferation, inflammation, and insulin secretion (Sims et al. 2013). These differential pathways affected by HFD between strains with differential susceptibility to IR and T2D are more likely to be relevant to T2D. Lastly, transgenerational effects of HFD have been examined and the findings from the transcriptomic studies corroborate with the increased susceptibility of offspring to metabolic diseases. For instance, male offspring from mothers fed HFD developed IR and their muscle tissues demonstrated dysregulation in cytokine signaling, starch and sucrose metabolism, inflammatory response, oxidative phosphorylation, mitochondrial matrix, and electron transport/uncoupling (Latouche et al. 2014). In a study of paternal HFD, female offspring were found to have perturbations in olfactory transduction and cell cycle processes in both adipose tissue and islet, along with additional tissue-specific processes such as ubiquitin-mediated proteolysis and mitochondria in adipose, and lipid metabolism, cell–cell signaling, and nervous systems development in islet (Ng et al. 2014).

In addition to HFD, high-sugar diets such as high fructose and sucrose have been more recently recognized as a potential risk of metabolic syndrome and diabetes independent of energy intake (Abdelmalek et al. 2012; Goran et al. 2013). A recent global study revealed that the consumption of high-fructose corn syrup explains a remarkable 20 % increase in T2D incidents [12]. Unlike glucose from which lipid synthesis is under cellular control through the energy-sensitive enzyme phosphofructokinase, fructose is metabolized by fructokinase to fructose-1-phosphate, which can bypass the enzymatic control by phosphofructokinase and be converted into fat [8, 9]. In addition, there is no negative feedback mechanism that regulates the phosphorylation of fructose to prevent hepatic ATP depletion (van den Berghe et al. 1977). Cellular ATP depletion can cause an arrest in protein synthesis and induce inflammatory and pro-oxidative changes (Cirillo et al. 2009). Consistent with these findings, transcriptomics studies (Table 1) indicate that high-fructose consumption promotes fatty acid biosynthesis, endoplasmic reticulum stress and stress-related kinase, apoptotic activity, and mitochondrial dysfunction in the liver (Chang et al. 2014). In a study of high-sucrose diet, Nojima et al. (2013) investigated liver transcriptome changes in a diabetes mouse model and found alterations in many pathways such as lipid/amino acid/steroid metabolism, cell cycle, transcription, apoptosis, and immune response. Although systematic interrogation of high-sugar diets is still lacking at present, comparison of the results with those from the HFD studies reveals similar pathway level perturbations in lipid metabolic pathways.

Another major macronutrient being studied in T2D is protein, mainly in the context of protein restriction in pregnancy which has been associated with hypertension, endothelial dysfunction, and blood glucose levels in the offspring (Gluckman et al. 2009; Sahajpal and Ashton 2003). Transcriptomic analysis of liver from a porcine model has revealed that maternal protein restriction diets alter a diverse set of pathways including cell cycle regulation, Wnt signaling, fatty acid elongation, steroid biosynthesis, glucocorticoid receptor signaling, mTOR signaling, VEGF signaling, and the complement system (Oster et al. 2012a, b).

Compared to macronutrients described above, micronutrients such as vitamins and minerals are required for growth in lower amounts and serve mainly as constituents of enzymes and metalloproteins. In particular, several micronutrients such as vitamin D and iron have been associated with glucose metabolism, insulin signaling, and β cell function, and high rates of micronutrient deficiencies have been observed in obese and T2D populations (Kaidar-Person et al. 2008a, b). Of the micronutrients examined in nutritranscriptomic studies, vitamin D is particularly interesting. The prevalence of vitamin D deficiency in obese individuals is over 80 % (Kaidar-Person et al. 2008a), and the presence of vitamin D receptors and the responsiveness of insulin gene expression to vitamin D in human pancreatic β cells suggest a role of vitamin D in β cell function and diabetes. Both animal model and human epidemiology studies also support a tight inverse relationship between vitamin D levels and β cell function or T2D prevalence (Via 2012). A recent transcriptomic study of islets responding to vitamin D implicated pathways including lipid metabolism, cell cycle, cellular assembly and organization, cellular function and maintenance, vitamin and mineral metabolism, and molecular transport (Wolden-Kirk et al. 2013). Moreover, under inflammatory conditions, vitamin D was found to prevent islet apoptosis and restored insulin secretion, accompanied by significant modulation of islet genes involved in immune response, chemotaxis, chemokine production, cell death, and pancreatic β cell function (Wolden-Kirk et al. 2014). Although clinical trials have failed to substantiate the beneficial role of vitamin D supplement in improving T2D (Via 2012), these islet transcriptional signatures nevertheless support an important role of vitamin D in T2D pathogenesis based on the relevance of the molecular pathways to T2D.

Another micronutrient of relevance to T2D is iron, because both iron overload and deficiency have been linked to impaired pancreatic function and glucose homeostasis. In a microarray analysis of rat pancreas with either iron deficiency or iron overload, genes involved in lipid transport and encoding pancreatitis-associated proteins were found to be significantly affected in both conditions, thus supporting their involvement in pancreatic functions and T2D (Coffey et al. 2014). These micronutrient studies, therefore, also point to a role of lipid metabolism/transport as a shared mechanism through which nutrients influence T2D.

## Nutriepigenomics in T2D

A potential source of the large-scale diet-induced transcriptomic alterations observed above could be epigenomic perturbations due to the critical role of the epigenome in gene expression regulation. Unlike genetic regulation of gene expression via DNA sequence variations, epigenomic regulatory mechanisms are void of DNA sequence changes. Epigenomics involves various types of modifications or organization of the DNA, including DNA methylation, histone modification (acetylation, methylation, phosphorylation, DP-ribosylation, and ubiquitination), and chromatin remodeling. They affect gene expression mainly by altering the accessibility of DNA to the transcriptional machinery. Noncoding RNAs such as microRNAs, small interfering RNAs, and lncRNAs are also important epigenetics regulators of gene expression through posttranscriptonal RNA degradation, transcriptional repression, chromatin modification, and histone and DNA methylation (Holoch and Moazed 2015). Importantly, the epigenome is more dynamic and responsive to external stimuli such as dietary changes. Several approaches have been developed over the past few years to measure the DNA methylome, including whole-genome bisulfite sequencing, restriction enzyme-enriched sequencing techniques, affinity-enrichment-based sequencing techniques, and DNA methylation arrays. The sequencing-based technologies have the capacity to simultaneously measure the methylation status of millions of DNA loci. The advantages and disadvantages of these technologies have been well discussed elsewhere (Heyn and Esteller 2012; Laird 2010). For histone modifications and chromatin organization, various sequencing-based methods, such as sequencing coupled with chromatin immunoprecipitation (ChIP-seq), formaldehyde-assisted isolation of regulatory elements (FAIRE-seq), and DNase-seq have become readily available, as reviewed in detail by others (Morozova and Marra 2008; Telese et al. 2013). RNA sequencing technologies described for mRNA profiling can be modified in the sample processing and RNA extraction steps for noncoding RNA studies (Pritchard et al. 2012).

The involvement of large-scale DNA methylation changes in T2D has been supported by a recent genome-wide screening, which identified 276 DNA loci with significant differential methylation in diabetic islets (Volkmar et al. 2012). Some of the differential methylation loci were accompanied by transcriptional changes in adjacent genes involved in β cell survival/function, cellular dysfunction, and stress adaptation (Volkmar et al. 2012). In another study comparing offspring from obese and diabetic mothers to offspring from lean mice through genome-wide assay of ~16,000 CpG methylation sites in the liver tissue, maternal obesity/T2D was found to trigger small but widespread methylation changes (Li et al. 2013). Surprisingly, the methylation changes were most concentrated at genes related to development, rather than genes affecting metabolism.

The detection of T2D-related DNA methylomic patterns makes it possible to compare these patterns with those affected by T2D-associated diets or nutritional factors to further understand the mechanistic connections of nutrition to T2D. The influences of dietary changes on epigenetic phenomena such as DNA methylation and various types of histone modifications have been extensively investigated, as recently summarized by Choi et al. (2013). However, the majority of nutriepigenomic studies have focused on candidate genes or loci rather than implementing the high-throughput genome-wide methodologies (Levian et al. 2014). In one recent study of male mice fed either folate poor or folate rich diets throughout their life, genome-wide DNA methylation analysis of the sperm showed that the two groups had differential methylation patterns at genes associated with many chronic diseases such as cancer and diabetes (Lambrot et al. 2013). In particular, it was found that folate deficiency is correlated with altered sperm DNA methylation of genes such as *Aff3*, *Nkx2*-*2*, and *Uts2*, which have been associated with diabetes. Another recent genome-wide profiling of open chromatin in mouse liver tissue using ChIP-seq and FAIRE-seq revealed that extensive changes in the liver chromatin structure of mice fed a HFD and that the differential chromatin regions varied depending on the strain of mice (Leung et al. 2014). The restructuring of the chromosomes occurred mostly at areas targeted by liver transcription factors and, not surprisingly, was correlated with changes in gene expression. The fact that epigenetic modification is most common at loci which regulate other genes provides an explanation for why epigenetic changes can have widespread and indirect effects.

MicroRNAs have also been associated with T2D as well as T2D-related traits such as adipogenesis, inflammatory responses, and insulin secretion and sensitivity (Dangwal et al. 2015; Romao et al. 2011; Ross and Davis 2014). In a recent small RNA sequencing study of human pancreatic islet and β cells, for example, miR-375 was identified as important for insulin secretion regulation and miR-107, miR-103, and let-7 were associated with insulin sensitivity (van de Bunt et al. 2013). As recently reviewed in detail by Ross and Davis (Ross and Davis 2014), many miRNAs such as let-7 were not only associated with T2D and cancer, but could be modulated by an array of dietary components such as curcumin, spinach, and polyunsaturated fatty acids. While the majority of noncoding RNA studies in T2D at present focus on miRNAs, lncRNAs have also recently gained recognition for their potential roles in pancreatic β cell function and glucose metabolism (Knoll et al. 2015; Kornfeld and Bruning 2014). Although genome-scale epigenomic studies of nutritional modulation are scarce at present, we envision rapid growth in this line of research in the future.

## Nutriproteomics in T2D

The functional consequences of transcriptomic and epigenomic changes are expected to be reflected in protein-level alterations. Proteomics that systematically examines protein species has recently been used in studies of diabetes, revealing an increasing number of enzymes and metabolic pathways related to the development of IR (Chowdhury et al. 2011; Sundsten and Ortsater 2009). Breker and Schuldiner (2014) recently summarized the revolutionary progresses in proteomics technologies in the past few years and described in detail the common proteomic assays such as one- and two-dimensional gel electrophoresis (2D-GE), protein chip, high-performance liquid chromatography (HPLC), and mass spectrometry (MS). These proteomics technologies provide us with information from static to dynamic measurements, from measuring protein abundance to obtaining translation levels and measuring posttranslational effects, and from population-level measurements to single cells (Breker and Schuldiner 2014). These approaches make monitoring protein biomarkers for physiological deregulation and the effects of nutrition much easier (Sauer and Luge 2015).

Like transcriptome, proteome studies in nutrigenomics have detected both well-studied and novel regulators and pathways. Chowdhury et al. (2011) gave a good review on nutrient excess and altered mitochondrial proteome and functions in diabetes that may contribute to neurodegeneration. Specifically, proteins involved in mitochondrial complex I–V, tricarboxylic acid (TCA) cycle, heat shock, fatty acid utilization were altered in diabetic sensory neurons (Akude et al. 2011). It was proposed that nutrient excess may trigger diminished NAD^+^/NADH ratio which in turn switches off AMP kinase and/or SIRT1 (surtuin 1) signaling cascade, leading to impaired expression or activity of peroxisome proliferator-activated receptor gamma coactivator-1 (PGC-1 alpha) and reduced mitochondrial activity in mouse neurons (Chowdhury et al. 2011). Recent studies of mouse livers reported that proteins involved in branched-chain amino acid degradation, fatty acid oxidation, TCA cycle, oxidative phosphorylation, and retinol metabolism were affected by HFD (Deng et al. 2010; Guo et al. 2013; Takamura et al. 2008). Furthermore, the association of proteins altered by fructose consumption with diabetes was examined in hamsters using a matrix-assisted laser desorption/ionization-based proteomics approach (Zhang et al. 2008). They found that the differentially expressed proteins were enriched in fructose catabolism, fatty acid metabolism, cholesterol and triglyceride metabolism, protein folding, and antioxidation. Agreeing with the nutritranscriptomic findings discussed before, these nutriproteomics studies found that high-fat and high-fructose diets both affect proteins involved lipid metabolism processes.

Proteomics altered by dietary fatty acid shifts was also investigated recently by Kawashima et al. (2013) using nano-HPLC–ESI–MS/MS. They compared the proteomics between two diets with varying amounts of polyunsaturated fatty acids—omega-3 and omega-6, and found that proteins involved in mitochondria, metabolic processes, and response to stimulus were perturbed in the liver tissue by the fatty acid shifts.

Like macronutrient imbalances described above, vitamin and mineral imbalances have also been found to exert profound effects on the activity and functions of proteins. This is not surprising given that micronutrients act as substrates, cofactors, and ligands of proteins directly responsible for catalytic or transport activities. For example, Ahmad et al. (2013) reported that maternal vitamin B12 deficiency induced differential levels of proteins involved in the regulation of amino acid, lipid, and carbohydrate metabolism as well enzymes in the β oxidation pathway in the liver of the offspring. The metabolic changes were proposed to be mediated by the PPAR signaling pathway.

While measuring protein abundance is helpful in capturing key pathways perturbed by nutritional imbalances, assessing protein posttranslational modifications is also valuable because they are critical to protein function/activity: They help shape the three-dimensional structures of proteins, modify activities of catalytic sites, and regulate binding partners and subcellular localization. Toward this end, Sverdlov et al. (2015) recently utilized HPLC to detect the oxidative posttranslational modification of mitochondrial complex II induced by high-fat/high-sucrose diet and found the posttranslational modification to be responsible for mitochondrial dysfunction. The methodological advances in proteomics will enable more comprehensive profiling of proteins and their modifications in nutriproteomics studies.

## Nutrimetabolomics in T2D

Diabetes is a metabolic disorder, and it has been shown that metabolites play important roles in IR and T2D (Ginter and Simko 2013). The rapidly developing discipline of metabolomics makes it possible to conduct high-resolution characterization of hundreds or thousands of metabolites from complex samples in a single measurement. Metabolomics has been widely adopted in pharmacology and toxicology to understand the effects of exogenous compounds on metabolic regulation but is rapidly rising in nutritional studies (Gibbons et al. 2015). At present, two analytical platforms are mainly used for metabolomics analyses: MS and nuclear magnetic resonance (NMR). Each platform has inherent advantages and disadvantages, such as the high reproducibility but a low sensitivity in NMR-based techniques compared with MS-based techniques (Gibbons et al. 2015; Gika et al. 2014). These metabolomics tools allow comprehensive measurements of key metabolites in signaling, receptor binding, translocation, and biochemical reaction pathways. In general, known biomarkers of diabetes such as sugar metabolites (e.g., 1,5-anhydroglucoitol), ketone bodies (e.g., 3-hydroxybutyrate), and branched-chain amino acids could be detected by various metabolomic approaches.

As summarized in Table 2, recent nutrimetabolomic studies of diabetes has revealed diet-specific changes in metabolites. For example, HFD increases lipid metabolites (such as phosphatidylcholines and fatty acids) but decreases lipid metabolism intermediates (such as various acyl carnitines) and the NAD^+^/NADH ratio, indicating decreased β-oxidation and abnormal lipid and energy metabolism (Kim et al. 2011). The levels of metabolites that are related to obesity-associated diseases, such as serotonin, pipecolic acid, uric acid, and branched-chain amino acid valine were altered by HFD (Kim et al. 2011). In addition, as reviewed by Lorraine Brennan et al., the branched-chain amino acids (BCAAs) were elevated in a variety of animal models fed with HFD (Gibbons et al. 2015). Studies of other diets or nutrients such as high fructose, low protein, vitamin B6 and vitamin D also revealed tissue- and nutrient-specific metabolite alterations (Table 2).

**Table 2:** Significant differential metabolites induced by diet imbalance in T2D-relevant tissues

Diet Imbalance	Tissues	Increased metabolites	Decreased metabolites
High-fat diet versus Chow (Kim et al. 2011)	Serum	Serotonin Phosphatidylcholine (PC) Pipecolic acid l-Carnitine Stearoylcarnitine Uric acid LysoPC (17:0), (18:0), (18:3) Valine, arginine, tyrosine, benzoic acid Pantothenic acid Phenylacetamide	Myristoylcarnitine Decanoylcarnitine Hexadecenoylcarnitine Vaccenylcarnitine Linoleylcarnitine LysoPE (18:2), (20:4) LysoPC (14:0), (15:0), (16:0), (16:1), (17:1), (18:1), (18:2), (19:0), (20:1), (20:4), (20:5)
	Liver	7-Ketodeoxycholic acid LysoPC (20:4) Monosaccharide Fatty acid Maltose-8TMS	L-Carnitine 3-Metyl-glutarylcarnitine LysoPC (16:1) *trans*-Palmitoleic acid-1TMS Tyrosine Glycerol-3TMS Glucose-5TMS NAD^+^/NADH
High-fructose diet versus Chow (Lin et al. 2011)	Blood plasma	Proline, methionine, proline, tryptophan, glutamine, glutamic acid, phenylalanine, leucine/isoleucine LysoPE (20:4), (18:1) LysoPC (20:4), (14:0), (20:5), (16:1)	α-/g-Linolenic acid (18:3) Docosahexaenoic acid (22:6) Eicosapentaenoic acid (20:5) Glycocholic acid
	Liver	LysoPC (22:5), (20:4), (18:1), (16:1), (20:4) Cytosine Oleic acid (18:1) Palmitoleic acid (16:1)	PC(18:4/20:2), (18:1/22:5), (20:2/16:0), (18:2/16:0) PE (22:6/16:0) Ergothioneine Malic acid Eicosapentaenoic acid (20: 5)
	Muscle	LysoPC (22:4) LysoPE (16:0) Adrenic acid (22:4) Docosapentaenoic acid (22:5)	PC(18:4/20:2), (18:1/22:5), (22:6/20:4), (22:5/16:1), (18:4/18:1), (20:0/15:0), (22:5/P-16:0), (24:1/15:0) Eicosapentaenoic acid (20:5)
High- versus low-protein diet (Rasmussen et al. 2012)	Diabetes Urine	Creatine Taurine TMAO	Citric acid
Vitamin B6 deficiency versus Chow (da Silva et al. 2013)	Blood plasma	Serine Cystathionine	Dimethylglycine Creatine Creatinine
Vitamin D deficiency versus Chow (Finkelstein et al. 2014)	Blood plasma	Pyridoxate Bilirubin Xylose cholate	Leukotrienes 1,2-propanediol Azelate Undecanedioate Sebacate Piperine

LysoPC, lysophosphatidylcholines; lysoPE, lysophosphatidylethanolamine; TMAO, trimethylamine-*N*-oxide

## Nutrimicrobiomics in T2D

The intestinal microbiome is a unique ecosystem and an essential mediator of metabolism by encoding enzymatic pathways that enable metabolism and synthesis of fatty acids and vitamins. They also contribute to the host immune development. In recent years, three major types of high-throughput sequencing-based technologies have become widely used to study whole communities of prokaryotes in many niches (Di Bella et al. 2013). The most commonly used one is amplicon sequencing, which amplifies and sequences specific variable regions of highly conserved genes (e.g., the 16S rRNA gene and the type 1 chaperonin gene *cpn60*) in order to determine which organisms are in a sample and how organisms differ between different conditions. In contrast to amplicon sequencing, shotgun metagenome sequencing and metatranscriptome sequencing aim to sequence all DNA and RNA in a sample to determine which genes are present and which genes are transcribed to what levels, respectively. Metagenome and metatranscriptome sequencing can detect not only changes in the microbiome spectrum, but also differentially expressed bacterial genes. However, there are numerous challenges including low coverage, difficulties in assembly, and potential ambiguous interpretation in these whole-genome technologies, as discussed in detail by Di Bella et al. (Di Bella et al. 2013).

Growing evidence supports that the microbiome in our body, especially in the gut, is altered in diabetes (Hartstra et al. 2015; Larsen et al. 2010; Qin et al. 2012). In a landmark metagenome-wide association study of 345 T2D patients and nondiabetic controls, Qin et al. (2012) identified ~60,000 diabetes-associated microbial gene markers using the gut microbial DNA. T2D patients were found to have decreasing abundance of butyrate-producing bacteria (*Clostridiales* sp. SS3/4, *Eubacterium rectale*, *Faecalibacterium prausnitzii*, *Roseburia intestinalis*, and *Roseburia inulinivorans*) and increasing opportunistic pathogens (*Bacteroides caccae*, *Clostridium hathewayi*, *Clostridium ramosum*, *Clostridium symbiosum*, *Eggerthella lenta*, and *Escherichia coli*) (Hartstra et al. 2015). Dietary composition and caloric intake appear to strongly and swiftly regulate microbial composition and function, but the underlying mechanisms have remained elusive.

As summarized in Table 3, the recent applications of microbiome sequencing approaches in nutritional studies ranging from high-caloric diets to food additives have significantly improved our understanding of the impact of dietary interventions on the microbiota. For instance, Wu et al. examined various diets in human subjects using 16S rDNA sequencing and found that microbiota enterotypes were strongly associated with long-term diets. In particular, protein and animal fat appear to favor *Bacteroides* enterotype defined by *Bacteroides, Alistipes*, and *Parabacteroides*, and carbohydrates promote *Prevotella* enterotype defined by *Prevotella*, *Paraprevotella* (phylum Bacteroidetes), and *Catenibacterium* (phylum Firmicutes) (Wu et al. 2011). The microbiota changes can subsequently influence nutrient acquisition, energy harvest, and diverse metabolic pathways in the host. For instance, the microbiome associated with obesity has been found to be more efficient in harvesting energy from the diet, alter host metabolic pathways such as fatty acid metabolism and lipid peroxidation, and activate inflammatory pathways, which are closely associated with IR and diabetes (Hartstra et al. 2015). On the other hand, butyrate-producing bacteria may protect individuals from T2D by inducing beneficial effects through the diverse actions of small chain fatty acids such as butyrate. As summarized by Hartstra et al. (2015), butyrate may play important roles in T2D prevention by enhancing mitochondrial activity, preventing metabolic endotoxemia, and activating intestinal gluconeogenesis. Butyrate likely achieves these effects through its interaction with histone deacetylases to regulate gene expression of key metabolic regulators such as PGC-1 alpha, a transcription coactivator associated with increased fatty acid oxidation and mitochondrial activity.

**Table 3:** Microbiota changes induced by nutritional modulation

Nutritional modulation	Species	Microorganisms changed in abundance		References
		Increase	Decrease	
Protein, fat	Human	*Bacteroides, Alistipes* and *Parabacteroides*	–	Wu et al. (2011)
Carbohydrate	Human	*Prevotella*, *Paraprevotella* (phylum Bacteroidetes) and *Catenibacterium* (phylum Firmicutes)	–	Wu et al. (2011)
Animal-based diet (meat, eggs, and cheeses)	Human	Alistipes, Bilophila and Bacteroidels	*Roseburia*, *Eubacterium rectale* and *Ruminococcus bromii*	David et al. (2014)
Maternal high-fat diet	Macaca fuscata	Ruminococcus and Dialister	*Campylobacter* spp. and *Helicobacter* spp.	Ma et al. (2014)
Parental high-fat diet	Mouse	The ratio of Firmicutes to Bacteroidetes	–	Myles et al. (2013)
High-fat diet	Mouse	The ratio of Firmicutes to Bacteroidetes, Ruminococcaceae and Rikenellaceae	*Bacteroidaceae, Clostridiales*, and *Provotellaceae*	Kim et al. (2012)
High-fat diet	Mouse	proportions of Firmicutes, Deferribacteres, and Proteobacteria	–	Walker et al. (2014)
High-protein diet	Rat	Lactobacillus	*Lachnospiraceae*	Pioli et al. (2013)
Potato fiber	Dog	Faecalibacterium	–	Panasevich et al. (2015)
Formula fed infants	Human	Ruminococcus	Lactobacillus	O’Sulliyan et al. (2013)
Saturated fat (from milk)	Human	*Bilophila wadsworthia*	–	Devkota et al. (2012)
Carbohydrate-rich diet	Human	Archaea *Methanobrevibacter*	–	Samuel and Gordon (2006)
Agrarian diet (carbohydrates, fiber, nonanimal protein)	Human	*Prevotella* and *Xylanibacter*	Firmicutes	De Filippo et al. (2010)
Fiber (starches or nonstarch polysaccharides)	Human	Proportions of *Ruminococcus bromii* and *Eubacterium rectale*	–	Albenberg and Wu (2014)
Milk oligosaccharides	Human	Bifidobacteria	–	Albenberg and Wu (2014)
Dietary emulsifiers (carboxymethyl-cellulose, polysobate-80)	Mouse	Mucolytic operational tazanomic units (e.g., *Ruminococcus gnavus*), Verrucomimicrobia phyla (e.g., *Akkermansia muciniphila*), proteobacteria	Bacteroidales	Chassaing et al. (2015)
Artificial sweeteners (saccharin, sucralose or aspartame)	Mouse	*Bacteroides* genus (*Bacteroides vulgatus, Bacteroides vulgatus), Orovidencia rettgeri, Parabacteroides distasonis, Staphylococcus aureus*	Clostridiales order *(Candidatus Arthromitus), Akkermansia muciniphila*	Suez et al. (2014)
Artificial sweeteners (saccharin, sucralose or aspartame)	Human	*Enterobacteriaceae* family, Deltaproteobacteria class, Actinobacteria phylum		Suez et al. (2014)

The strong influence of diets on gut microbiota points to potential therapeutic avenues through modulating bacterial metabolites, fecal transplantation, and probiotics. Indeed, oral administration of butyrate or fecal transplantation has been shown to improve insulin sensitivity, increase energy expenditure, and reverse metabolic syndrome in mice (Chassaing et al. 2015; Gao et al. 2009; Suez et al. 2014). In a double-blind randomized controlled trial, insulin-resistant males with metabolic syndrome received feces infusion from lean donors showed significant improvement in muscle insulin sensitivity, increased intestinal microbial diversity, and increased butyrate-producing bacteria, such as *Roseburia* in the feces and *Eubacterium halii* in the small intestine (Vrieze et al. 2012).

## Multi-omics integration in T2D

Through the above review of the applications of individual omics technologies in T2D research, we have summarized the various genes, pathways, epigenetic alterations, proteins, metabolites, and gut bacteria species that are affected by nutritional variations and potentially important for T2D pathogenesis. However, how these different levels of molecular signals piece together in the T2D puzzle is still unclear, primarily due to the focus of these studies on individual omics and hence the limited availability of systems-level data for a given diet or nutrient. In fact, even the basic associations between DNA methylation, gene expression, and metabolite profiles throughout the human genome remain poorly described, and only modest correlations have been observed between proteins and their corresponding mRNAs (Olsson et al. 2014; Petersen et al. 2014). Therefore, there is an urgent need for systems and integrative nutrigenomics to delineate the information flow from dietary or nutritional changes to the omics alterations observed. Integrative genomics will not only provide comprehensive knowledge about the information flow from DNA to gene transcript to protein to metabolite based on the central dogma of molecular biology, but also expand the central dogma by incorporating the microbiome (considered to be the second genome) and epigenomics (responsible for mediating environmental responses).

Toward this end, several recent studies have been carried out to characterize and integrate multi-layered nutri-omics data to better understand the systematic effects of nutritional variations. Wu et al. (2014) quantified the transcriptome, metabolome, and targeted proteomics of the liver tissues from 40 mouse strains fed on a chow diet or HFD and focused on 192 metabolic genes. The integration of the multi-layered information allowed detection of dozens of genetic loci, termed quantitative trait loci (QTLs), for transcripts, proteins, and metabolites involved in mitochondrion function and general metabolism. Interestingly, many of the QTLs are diet dependent, supporting gene by diet interactions in gene and protein regulations. Corroborating with the previously observed modest correlation between proteins and gene transcripts, genetic regulation of gene expression and that of proteins appeared to be mostly distinct as demonstrated by unique mapping of 80 % of QTLs to either proteins or transcripts but not both. These results support complex genetic regulation of molecular phenotypes that does not follow a simple linear model of one DNA locus to one gene to one protein. By connecting the protein QTLs with diabetic phenotypes, proteins Dhtkd1 and Ndufa4 were identified as candidates for glucose regulation, and Nnt for insulin secretion. Further investigation of Dhtkd1, a mitochondrial protein involved in lysine metabolism, revealed that it may regulate insulin sensitivity and glucose levels through a metabolite 2-aminoadipate (2-AA) which has an overlapping QTL with Dhtkd1. This novel insight would not have been possible without the integration of multi-layered omics data in the same study.

Even if comprehensive omics profiling can be achieved, the dynamic nature of dietary patterns and the subsequent molecular changes is often missed in traditional nutri-omics studies. In a unique study focusing on the circadian patterns of liver transcriptome and metabolome, Eckel-Mahan et al. (2013) identified circadian clock as one of the central mechanisms that mediate the HFD-induced large-scale metabolic and transcriptional reprogramming in liver. Specifically, they found that HFD interfered with CLOCK:BMAL1 recruitment to chromatin and induced *de novo* oscillation of PPARg-target genes. As a result, HFD feeding induced phase changes (mostly advanced peak time) of oscillations of metabolites of nucleotide, carbohydrates, and cofactors and vitamins, disrupted oscillations of metabolites of xenobiotics, amino acids, and nicotinamide adenine dinucleotide (NAD^+^), and promoted oscillations of lipid metabolites. At the transcriptome level, oscillation of genes involved in endocytosis, lysosome, proteolysis, insulin signaling, bile acid, and fatty acid synthesis was abolished, whereas that of glycerophospholipid metabolism, antigen processing and presentation, N-glycan biosynthesis, and protein processing in ER was induced. Coherence was observed between the metabolome and transcriptome, especially within amino acid metabolic pathways of cysteine, methionine, S-adenosylmethionine (SAM), and taurine. These changes were accompanied with de novo oscillation of genes with methyltransferase activities which may subsequently affect the epigenome. Compared to most other omics studies, the deep mechanistic insights from this study are in debt to its unique design that incorporates dynamic information of diverse molecular phenotypes. In support of the findings from this study, an increasing number of omics studies indicate that most dietary imbalances distort circadian cycle, which leads to aberrations in metabolism and contributes to obesity, IR, and others phenotypes consistent with diabetes (Kalsbeek et al. 2014; Lin et al. 2015).

Increasing amount of evidence supports that dietary responses can be dependent on the genetic background. To move beyond examining dietary effect on a single genetic background at a time and march into systematic interrogation of gene by diet interaction, the Lusis group has developed a rich systems genetic resource—the Hybrid Mouse Diversity Panel (HMDP)—that involves more than one hundred inbred or recombinant inbred strains of mice fed on either a chow or a high-fat, high-sucrose diet (Bennett et al. 2010; Ghazalpour et al. 2012). Using HMDP, Parks et al. (2013, 2015) integrated genetics, microbiome, gene expression from adipose tissue, and dietary information to study obesity in one study and leveraged genetics, gene expression, metabolites, gender, and diet information to unravel novel biology of IR in another. These studies revealed novel genetic loci, genes, metabolites, and microbiome species important for T2D-related traits. In the obesity study, strong genetic control of body fat set-point as well as microbiome plasticity was unraveled, and multiple genetic loci were identified for obesity traits and dietary responses (e.g., *Sptlc3*, *Klf14*, *Degs1, Npc, Cbr1,* and amylases) (Parks et al. 2013). In the IR study, they identified gender-specific genetic loci controlling IR variation as well as genetic loci for plasma metabolites and gene expression in the liver and adipose tissues. A total of 15 genetic loci were detected for IR, and a novel gene, *Agpat5*, was experimentally validated (Parks et al. 2015). These powerful systems genetics studies not only provide valuable data sources and biological insights but offer opportunities for development of novel approaches to integrate the multi-layered omics datasets.

## Conclusions

**Fig. 2:**
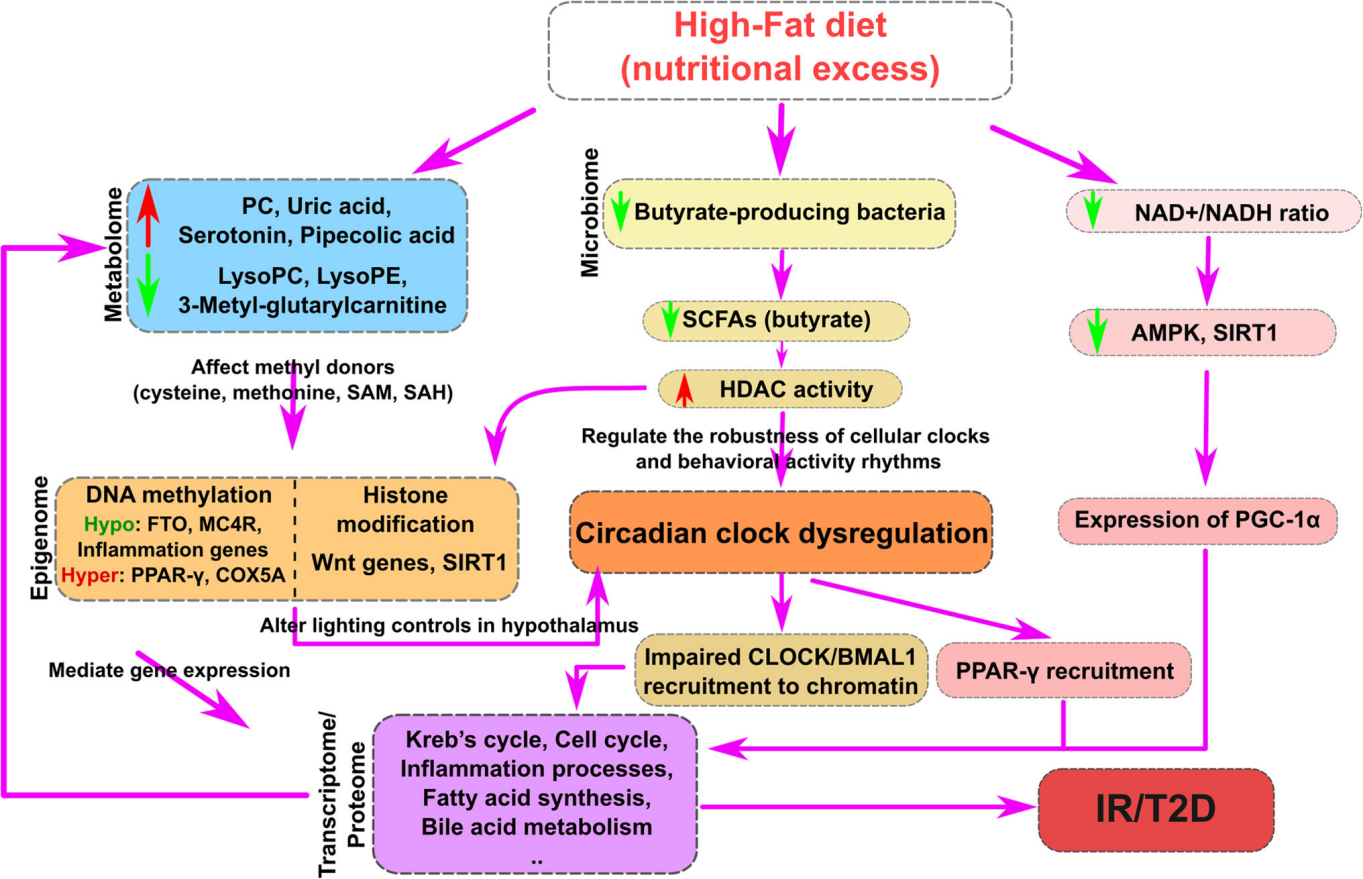
Potential mechanisms underlying high-fat-diet-induced diabetes based on recent nutritional systems biology studies. High-fat diet can affect metabolites (*left branch*), microbiota (*middle*), and NAD^+^/NADH ratio (*right*). *Left branch*: The perturbed metabolites may affect methyl donors such as cysteine, methionine, SAM, and SAH, leading to changes in DNA methylation. Altered DNA methylation regulates gene expression through multiple mechanisms, such as promoter and gene body methylation. *Middle branch*: Butyrate-producing bacteria have been found to be decreased in gut microbiota, leading to lower levels of short-chain fatty acids (SCFAs) such as butyrate, which could modulate histone deacetylase (HDAC) activities to induce histone modifications and chromatin structural changes. Epigenomic changes may directly alter transcriptional activities or indirectly by reshaping the circadian rhythm including impaired CLOCK/BMAL1 recruitment to chromatin and induction of PPAR-γ recruitment. *Right branch*: Decreased NAD^+^/NADH ratio by HFD can switch off AMPK and SIRT1 signaling, leading to downregulation of PGC-1 and subsequent mitochondria dysfunction. The upstream regulatory mechanisms depicted from all three branches will trigger in perturbations of various biological processes such as lipid metabolism, Krebs cycle, fatty acid synthesis, oxidative phosphorylation, cell cycle, and inflammatory responses that lead to insulin resistance and compromised β cell functions that are primary features of T2D

Our review of nutritional systems biology studies in T2D testifies to the power of omics technologies in discovering novel biomarkers that can be used to diagnose, predict, and monitor the progress of diabetes as well as in unraveling important mechanistic insights for developing preventative and therapeutic strategies. The omics studies summarized here have revealed a remarkably broad impact of dietary imbalance on the molecular systems and a highly complex regulatory network that connect the nutritional perturbations to T2D. As exemplified in Fig. 2 based on findings from HFD studies, the available evidence supports that deleterious shifts in dietary components lead to major metabolomics changes and promote gut microbiomic dysbiosis, which can further exacerbate metabolomic dysregulation. Alterations in key metabolites, some of which are capable of modifying methyl donors or key histone modification enzymes, can modify the epigenome and perturb circadian rhythm to promote reprogramming of the transcriptome and proteome. These reprograming events eventually lead to disruptions in the diversity, quantity, as well as oscillation patterns of genes and proteins involved in key metabolic pathways and immune and inflammatory processes that are important for T2D development.

## Remaining challenges and future directions

Compared to other scientific disciplines such as molecular genetics that have experienced evolutionary advances in the past few decades, studies of how food and nutrition interact with our internal body systems to affect health and disease are still in its infancy and our journey remains long due to multiple challenges. First, a comprehensive list of dietary and nutritional components that are relevant to T2D is not yet available. To date, most T2D studies focus on macronutrients, such as high fat, high carbohydrate (in particular dietary sugars) and low protein, and micronutrients such as Vitamin D, magnesium, and iron. Further exploration and refinement of the dietary components posing risk to T2D are warranted. For instance, a recent study of 2422 normoglycemic individuals of which 201 developed diabetes identified five branched-chain and aromatic amino acids, including isoleucine, leucine, valine, tyrosine, and phenylalanine, to be significantly associated with incident diabetes (Wang et al. 2011). Comprehensive amino acid profiling will also provide molecular insights into T2D pathogenesis.

Second, the remarkable complexity in dietary composition, tissue-specific responses, and the dynamic nature of dietary response makes it difficult to collect all relevant information by any single research group. Specifically, it is likely that not only the absolute quantities of macronutrients and micronutrients matter, but the ratios between nutrients are also critical (Asif 2014). The complexity of diets makes it arduous to test all possible combinations of nutrients with varying composition and may contribute to inconsistencies across nutritional studies due to the variability of diet composition. Moreover, the tissue and cell type-specific effects of nutrition demand comprehensive profiling of all disease-relevant tissues, which remains cost prohibitive. Furthermore, unlike genetics which is relatively static, dietary intake is a dynamic process and biological responses to nutritional imbalance are also diverse and dynamic, which requires close examination of multiple time points and long-term follow-up. The challenges call for coordinated efforts in the nutritional scientific community to join force and systematically characterize diets and nutrition in much a similar way as the consortium-based genetic studies that have been highly successful in the past few years.

Third, certain omics areas still face technological challenges. For example, short-read RNA-seq has become the de facto standard in transcriptome analysis, but poses challenge for precise reconstruction of transcript structures due to the complexity of higher-eukaryotic transcriptomes (Tilgner et al. 2014). Future applications of long-read sequencing in nutrition studies will facilitate transcriptome reconstruction and functional annotations. As discussed above, novel technologies for systematic analysis of proteomics have emerged, yet the application of these advanced tools in nutriproteomics is still in its infancy. Recently, Akhilesh Pandey et al. applied high-resolution Fourier transform mass spectrometry to draw a draft map of the human proteome, capturing ~17,000 proteins representing 84 % of the total annotated protein-coding genes in humans (Kim et al. 2014), which we believe will be valuable in evaluating the alterations of proteomic profiles under diverse nutrient conditions. In addition, the nutriproteome also requires studying protein dynamics at single-cell resolution, because it has become clear that populations of cells, even genetically identical ones, show high variability in response to nutrition perturbations (Sauer and Luge 2015).

Lastly, although our ability to generate multi-layer molecular data has significantly improved over the past few decades, we are facing an ever-increasing challenge in efficient and accurate data integration. Recently, various network modeling approaches have been developed and utilized to alleviate this pressing concern (Bordbar and Palsson 2012; de Graaf et al. 2009; Levian et al. 2014; Mc Auley et al. 2012; Meng et al. 2013; Zhao and Huang 2011). However, their efficiency in integrating the exploding data sources and their ability to accurately model dynamic systems remain limited. More advanced methodologies are urgently needed.

All these challenges demand coordinated efforts and broad collaborations across disciplines in order to achieve systematic characterization and understanding of the role of diverse types of food and nutrition, the most fundamental elements of living beings, in maintaining or compromising health. Such systems-level comprehensive understanding will have the potential to transform medicine from traditional symptom-oriented diagnosis and treatment of diseases toward disease prevention and early diagnostics. An example moving into this direction is the integrated Personal Omic Profiling (iPOP) system developed by the Snyder group at Stanford (Chen et al. 2012b). By tracing the dynamic personal genome, it is possible to observe molecular changes accompanying external variations such as lifestyle and dietary modification, and use the molecular information to predict health consequences. Such systems will better assist in health care in many ways such as early and accurate diagnosis and disease prevention. Studies that have investigated gene–lifestyle interactions in T2D have suggested that the biological effects of genetic predisposition may be partially or nearly completely abolished by a healthy lifestyle or lifestyle modifications (Temelkova-Kurktschiev and Stefanov 2012). With the help of nutritional systems biology, we will be able to obtain comprehensive molecular signature maps of dietary components posing T2D risks. When such maps are coupled with a personalized iPOP system, it is possible to pinpoint nutritional factors that can reverse or normalize the risk molecular profiles specific to a particular individual to achieve truly personalized health care. A recent study demonstrating that dietary Jerusalem artichoke (Helianthus tuberosus) reversed both the gene expression pattern and T2D-related phenotypes induced by high fructose (Chang et al. 2014) supports this concept and marks an inch toward the reality of diet-based treatment of T2D. Although directly relevant, human studies face difficulties in accessing internal tissues, which limit their ability to obtain systems-level biological insights. Animal models studies, on the other hand, can facilitate mechanistic insights but are limited by their direct translational potential. Therefore, coupling animal model studies and human investigations like the iPOP system in nutritional systems biology is necessary to enable not only rapid discovery but also timely translation into humans.

## References

[CR1] Abdelmalek MF (2012). Higher dietary fructose is associated with impaired hepatic adenosine triphosphate homeostasis in obese individuals with type 2 diabetes. Hepatology.

[CR2] Ahmad S (2013). PPAR signaling pathway is a key modulator of liver proteome in pups born to vitamin B-12 deficient rats. J Proteomics.

[CR3] Akude E, Zherebitskaya E, Chowdhury SK, Smith DR, Dobrowsky RT, Fernyhough P (2011). Diminished superoxide generation is associated with respiratory chain dysfunction and changes in the mitochondrial proteome of sensory neurons from diabetic rats. Diabetes.

[CR4] Albenberg LG, Wu GD (2014). Diet and the intestinal microbiome: associations, functions, and implications for health and disease. Gastroenterology.

[CR5] Alberti KGM, Zimmet P (2013). Epidemiology: global burden of disease—Where does diabetes mellitus fit in?. Nat Rev Endocrinol.

[CR6] Ardisson Korat AV, Willett WC, Hu FB (2014). Diet, lifestyle, and genetic risk factors for type 2 diabetes: a review from the nurses’ health study, nurses’ health study 2, and health professionals’ follow-up study. Curr Nutr Rep.

[CR7] Asif M (2014). The prevention and control the type-2 diabetes by changing lifestyle and dietary pattern. J Educ Health Promot.

[CR8] Atkins RC, Zimmet P, St I-IWKD (2010). DIABETES diabetic kidney disease: act now or pay later. Nat Rev Nephrol.

[CR9] Barbosa-Sampaio HC (2013). Nupr1 deletion protects against glucose intolerance by increasing beta cell mass. Diabetologia.

[CR10] Bennett BJ (2010). A high-resolution association mapping panel for the dissection of complex traits in mice. Genome Res.

[CR11] Bordbar A, Palsson BO (2012). Using the reconstructed genome-scale human metabolic network to study physiology and pathology. J Intern Med.

[CR12] Breker M, Schuldiner M (2014). The emergence of proteome-wide technologies: systematic analysis of proteins comes of age. Nat Rev Mol Cell Biol.

[CR13] Capozzi F, Bordoni A (2013). Foodomics: a new comprehensive approach to food and nutrition. Genes Nutr.

[CR14] Cech TR, Steitz JA (2014). The noncoding RNA revolution-trashing old rules to forge new ones. Cell.

[CR15] Chang W-C, Jia H, Aw W, Saito K, Hasegawa S, Kato H (2014). Beneficial effects of soluble dietary *Jerusalem artichoke* (*Helianthus tuberosu*s) in the prevention of the onset of type 2 diabetes and non-alcoholic fatty liver disease in high-fructose diet-fed rats. Br J Nutr.

[CR16] Chassaing B, Koren O, Goodrich JK, Poole AC, Srinivasan S, Ley RE, Gewirtz AT (2015). Dietary emulsifiers impact the mouse gut microbiota promoting colitis and metabolic syndrome. Nature.

[CR17] Chen L, Magliano DJ, Zimmet PZ (2012). The worldwide epidemiology of type 2 diabetes mellitus-present and future perspectives. Nat Rev Endocrinol.

[CR18] Chen R (2012). Personal omics profiling reveals dynamic molecular and medical phenotypes. Cell.

[CR19] Choi SW, Claycombe KJ, Martinez JA, Friso S, Schalinske KL (2013). Nutritional epigenomics: a portal to disease prevention advances. Nutrition.

[CR20] Chowdhury SKR, Dobrowsky RT, Femyhough P (2011). Nutrient excess and altered mitochondrial proteome and function contribute to neurodegeneration in diabetes. Mitochondrion.

[CR21] Cirillo P (2009). Ketohexokinase-dependent metabolism of fructose induces proinflammatory mediators in proximal tubular cells. J Am Soc Nephrol.

[CR22] Coffey R, Nam H, Knutson MD (2014). Microarray analysis of rat pancreas reveals altered expression of Alox15 and regenerating islet-derived genes in response to iron deficiency and overload. PLoS ONE.

[CR23] Cui J, Le G, Yang R, Shi Y (2009). Lipoic acid attenuates high fat diet-induced chronic oxidative stress and immunosuppression in mice jejunum: a microarray analysis. Cell Immunol.

[CR24] da Silva VR (2013). Metabolite profile analysis reveals functional effects of 28-day vitamin B-6 restriction on one-carbon metabolism and tryptophan catabolic pathways in healthy men and women. J Nutr.

[CR25] Dangwal S (2015). Impairment of wound healing in patients with type 2 diabetes mellitus influences circulating MicroRNA patterns via inflammatory cytokines. Arterioscl Throm Vas.

[CR26] David LA (2014). Diet rapidly and reproducibly alters the human gut microbiome. Nature.

[CR27] De Filippo C (2010). Impact of diet in shaping gut microbiota revealed by a comparative study in children from Europe and rural Africa. Proc Natl Acad Sci.

[CR28] de Fourmestraux V (2004). Transcript profiling suggests that differential metabolic adaptation of mice to a high fat diet is associated with changes in liver to muscle lipid fluxes. J Biol Chem.

[CR29] de Graaf AA (2009). Nutritional systems biology modeling: from molecular mechanisms to physiology. PLoS Comput Biol.

[CR30] de Wilde J (2009). An 8-week high-fat diet induces obesity and insulin resistance with small changes in the muscle transcriptome of C57BL/6J mice. J Nutrigenetics Nutrigenomics.

[CR31] Dearden L, Balthasar N (2014). Sexual dimorphism in offspring glucose-sensitive hypothalamic gene expression and physiological responses to maternal high-fat diet feeding. Endocrinology.

[CR32] Deng WJ, Nie S, Dai J, Wu JR, Zeng R (2010). Proteome, phosphoproteome, and hydroxyproteome of liver mitochondria in diabetic rats at early pathogenic stages. Mol Cell Proteomics.

[CR33] Devkota S (2012). Dietary-fat-induced taurocholic acid promotes pathobiont expansion and colitis in Il10-/-mice. Nature.

[CR34] Di Bella JM, Bao YG, Gloor GB, Burton JP, Reid G (2013). High throughput sequencing methods and analysis for microbiome research. J Microbiol Meth.

[CR35] Ding Y (2013). DNA hypomethylation of inflammation-associated genes in adipose tissue of female mice after multigenerational high fat diet feeding. Int J Obes (Lond).

[CR36] Donath MY, Shoelson SE (2011). Type 2 diabetes as an inflammatory disease. Nat Rev Immunol.

[CR37] Eckel-Mahan KL (2013). Reprogramming of the circadian clock by nutritional challenge. Cell.

[CR38] Eid J (2009). Real-time DNA sequencing from single polymerase molecules. Science.

[CR39] Finkelstein JL, Pressman EK, Cooper EM, Kent TR, Bar HY, O’Brien KO (2014). Vitamin D status affects serum metabolomic profiles in pregnant adolescents. Reprod Sci.

[CR40] Gao Z (2009). Butyrate improves insulin sensitivity and increases energy expenditure in mice. Diabetes.

[CR41] Ghazalpour A (2012). Hybrid mouse diversity panel: a panel of inbred mouse strains suitable for analysis of complex genetic traits. Mamm Genome.

[CR42] Gibbons H, O’Gorman A, Brennan L (2015). Metabolomics as a tool in nutritional research. Curr Opin Lipidol.

[CR43] Gika HG, Theodoridis GA, Plumb RS, Wilson ID (2014). Current practice of liquid chromatography–mass spectrometry in metabolomics and metabonomics. J Pharm Biomed Anal.

[CR44] Ginter E, Simko V (2013). Type 2 diabetes mellitus, pandemic in 21st century. Adv Exp Med Biol.

[CR45] Gluckman PD, Hanson MA, Buklijas T, Low FM, Beedle AS (2009). Epigenetic mechanisms that underpin metabolic and cardiovascular diseases. Nat Rev Endocrinol.

[CR46] Goran MI, Ulijaszek SJ, Ventura EE (2013). High fructose corn syrup and diabetes prevalence: a global perspective. Glob Pub Health.

[CR47] Guo YR (2013). Quantitative proteomic and functional analysis of liver mitochondria from high fat diet (HFD) diabetic mice. Mol Cell Proteomics.

[CR48] Hartstra AV, Bouter KEC, Backhed F, Nieuwdorp M (2015). Insights into the role of the microbiome in obesity and type 2 diabetes. Diabetes Care.

[CR49] Heyn H, Esteller M (2012). DNA methylation profiling in the clinic: applications and challenges. Nat Rev Genet.

[CR50] Holoch D, Moazed D (2015). RNA-mediated epigenetic regulation of gene expression. Nat Rev Genet.

[CR51] Hu FB (2011). Globalization of diabetes: the role of diet, lifestyle, and genes. Diabetes Care.

[CR52] Inoue M (2005). Increased expression of PPAR gamma in high fat diet-induced liver steatosis in mice. Biochem Biophys Res Commun.

[CR53] Ioannidis JPA, Tarone R, McLaughlin JK (2011). The false-positive to false-negative ratio in epidemiologic studies. Epidemiology.

[CR54] Kaidar-Person O, Person B, Szomstein S, Rosenthal RJ (2008). Nutritional deficiencies in morbidly obese patients: a new form of malnutrition? Part A: vitamins. Obes Surg.

[CR55] Kaidar-Person O, Person B, Szomstein S, Rosenthal RJ (2008). Nutritional deficiencies in morbidly obese patients: a new form of malnutrition? Part B: minerals. Obes Surg.

[CR56] Kalsbeek A, la Fleur S, Fliers E (2014). Circadian control of glucose metabolism. Mol Metab.

[CR57] Kastorini CM, Panagiotakos DB (2009). Dietary patterns and prevention of type 2 diabetes: from research to clinical practice; a systematic review. Curr Diabetes Rev.

[CR58] Kawashima Y, Singh A, Kodera Y, Matsumoto H (2013). Nutritional proteomics: investigating molecular mechanisms underlying the health beneficial effect of functional foods. Funct Foods Health Dis.

[CR59] Kim S, Sohn I, Ahn JI, Lee KH, Lee YS (2004). Hepatic gene expression profiles in a long-term high-fat diet-induced obesity mouse model. Gene.

[CR60] Kim HJ (2011). Metabolomic analysis of livers and serum from high-fat diet induced obese mice. J Proteome Res.

[CR61] Kim KA, Gu W, Lee IA, Joh EH, Kim DH (2012). High fat diet-induced gut microbiota exacerbates inflammation and obesity in mice via the TLR4 signaling pathway. PLoS ONE.

[CR62] Kim MS (2014). A draft map of the human proteome. Nature.

[CR63] Knoll M, Lodish HF, Sun L (2015). Long non-coding RNAs as regulators of the endocrine system. Nat Rev Endocrinol.

[CR64] Kornfeld JW, Bruning JC (2014). Regulation of metabolism by long, non-coding RNAs. Front Genet.

[CR65] Koza RA (2006). Changes in gene expression foreshadow diet-induced obesity in genetically identical mice. PLoS Genet.

[CR66] Laird PW (2010). Principles and challenges of genomewide DNA methylation analysis. Nat Rev Genet.

[CR67] Lambrot R (2013). Low paternal dietary folate alters the mouse sperm epigenome and is associated with negative pregnancy outcomes. Nat Commun.

[CR68] Larsen N (2010). Gut microbiota in human adults with type 2 diabetes differs from non-diabetic adults. PLoS ONE.

[CR69] Latouche C (2014). Maternal overnutrition programs changes in the expression of skeletal muscle genes that are associated with insulin resistance and defects of oxidative phosphorylation in adult male rat offspring. J Nutr.

[CR70] Lee AK (2010). Effect of high-fat feeding on expression of genes controlling availability of dopamine in mouse hypothalamus. Nutrition.

[CR71] Lee RK, Hittel DS, Nyamandi VZ, Kang L, Soh J, Sensen CW, Shearer J (2012). Unconventional microarray design reveals the response to obesity is largely tissue specific: analysis of common and divergent responses to diet-induced obesity in insulin-sensitive tissues. Appl Physiol Nutr Metab.

[CR72] Leung A (2014). Open chromatin profiling in mice livers reveals unique chromatin variations induced by high fat diet. J Biol Chem.

[CR73] Levian C, Ruiz E, Yang X (2014). The pathogenesis of obesity from a genomic and systems biology perspective. Yale J Biol Med.

[CR74] Li CC (2013). Maternal obesity and diabetes induces latent metabolic defects and widespread epigenetic changes in isogenic mice. Epigenetics.

[CR75] Lin SH, Yang Z, Liu HD, Tang LH, Cai ZW (2011). Beyond glucose: metabolic shifts in responses to the effects of the oral glucose tolerance test and the high-fructose diet in rats. Mol BioSyst.

[CR76] Lin LL, Huang HC, Juan HF (2015). Circadian systems biology in Metazoa. Briefings Bioinform.

[CR77] Ma J (2014). High-fat maternal diet during pregnancy persistently alters the offspring microbiome in a primate model. Nat Commun.

[CR78] Matsui N (2005). Ingested cocoa can prevent high-fat diet-induced obesity by regulating the expression of genes for fatty acid metabolism. Nutrition.

[CR79] Mc Auley MT, Wilkinson DJ, Jones JJL, Kirkwood TBL (2012). A whole-body mathematical model of cholesterol metabolism and its age-associated dysregulation. BMC Syst Biol.

[CR80] Meng Q, Makinen VP, Luk H, Yang X (2013). Systems biology approaches and applications in obesity, diabetes, and cardiovascular diseases. Curr Cardiovasc Risk Rep.

[CR81] Miller AM (2013). MiR-155 has a protective role in the development of non-alcoholic hepatosteatosis in mice. PLoS ONE.

[CR82] Morine MJ (2013). Network analysis of adipose tissue gene expression highlights altered metabolic and regulatory transcriptomic activity in high-fat-diet-fed IL-1RI knockout mice. J Nutr Biochem.

[CR83] Morozova O, Marra MA (2008). Applications of next-generation sequencing technologies in functional genomics. Genomics.

[CR84] Myles IA, Fontecilla NM, Janelsins BM, Vithayathil PJ, Segre JA, Datta SK (2013). Parental dietary fat intake alters offspring microbiome and immunity. J Immunol.

[CR85] Ng SF, Lin RC, Maloney CA, Youngson NA, Owens JA, Morris MJ (2014). Paternal high-fat diet consumption induces common changes in the transcriptomes of retroperitoneal adipose and pancreatic islet tissues in female rat offspring. FASEB J.

[CR86] Nojima K, Sugimoto K, Ueda H, Babaya N, Ikegami H, Rakugi H (2013). Analysis of hepatic gene expression profile in a spontaneous mouse model of type 2 diabetes under a high sucrose diet. Endocr J.

[CR87] Oh TS, Yun JW (2012). DNA microarray analysis reveals differential gene expression in the soleus muscle between male and female rats exposed to a high fat diet. Mol Biol Rep.

[CR88] Olsson AH (2014). Genome-wide associations between genetic and epigenetic variation influence mRNA expression and insulin secretion in human pancreatic islets. PLoS Genet.

[CR89] Oster M, Murani E, Metges CC, Ponsuksili S, Wimmers K (2012). A low protein diet during pregnancy provokes a lasting shift of hepatic expression of genes related to cell cycle throughout ontogenesis in a porcine model. BMC Genomics.

[CR90] Oster M, Murani E, Metges CC, Ponsuksili S, Wimmers K (2012). A low protein diet during pregnancy provokes a lasting shift of hepatic expression of genes related to cell cycle throughout ontogenesis in a porcine model. BMC Genom.

[CR91] O’Sulliyan A, He X, McNiven EMS, Haggarty NW, Lonnerdal B, Slupsky CM (2013). Early diet impacts infant rhesus gut microbiome, immunity, and metabolism. J Proteome Res.

[CR92] Ozsolak F, Milos PM (2011). RNA sequencing: advances, challenges and opportunities. Nat Rev Genet.

[CR93] Panagiotou G, Nielsen J (2009). Nutritional systems biology: definitions and approaches. Annu Rev Nutr.

[CR94] Panasevich MR (2015). Modulation of the faecal microbiome of healthy adult dogs by inclusion of potato fibre in the diet. Br J Nutr.

[CR95] Parks BW (2013). Genetic control of obesity and gut microbiota composition in response to high-fat, high-sucrose diet in mice. Cell Metab.

[CR96] Parks BW (2015). Genetic architecture of insulin resistance in the mouse. Cell Metab.

[CR97] Patsouris D, Reddy JK, Muller M, Kersten S (2006). Peroxisome proliferator-activated receptor alpha mediates the effects of high-fat diet on hepatic gene expression. Endocrinology.

[CR98] Petersen AK (2014). Epigenetics meets metabolomics: an epigenome-wide association study with blood serum metabolic traits. Hum Mol Genet.

[CR99] Pioli K, Barbieri C, Cann I, Mackie R, Beverly J (2013). High protein diet reduces food intake and adiposity and alters GI microbiome. FASEB J.

[CR100] Primeaux SD, Braymer HD, Bray GA (2013). High fat diet differentially regulates the expression of olfactory receptors in the duodenum of obesity-prone and obesity-resistant rats. Dig Dis Sci.

[CR101] Pritchard CC, Cheng HH, Tewari M (2012). MicroRNA profiling: approaches and considerations. Nat Rev Genet.

[CR102] Qin JJ (2012). A metagenome-wide association study of gut microbiota in type 2 diabetes. Nature.

[CR103] Rasmussen LG (2012). Assessment of the effect of high or low protein diet on the human urine metabolome as measured by NMR. Nutrients.

[CR104] Rivera CM, Ren B (2013). Mapping human epigenomes. Cell.

[CR105] Roat R (2014). Alterations of pancreatic islet structure, metabolism and gene expression in diet-induced obese C57BL/6J Mice. PLoS ONE.

[CR106] Romao JM, Jin WW, Dodson MV, Hausman GJ, Moore SS, Guan LL (2011). MicroRNA regulation in mammalian adipogenesis. Exp Biol Med.

[CR107] Ross SA, Davis CD (2014). The emerging role of microRNAs and nutrition in modulating health and disease. Annu Rev Nutr.

[CR108] Sahajpal V, Ashton N (2003). Renal function and angiotensin AT(1) receptor expression in young rats following intrauterine exposure to a maternal low-protein diet. Clin Sci.

[CR109] Samuel BS, Gordon JI (2006). A humanized gnotobiotic mouse model of host–archaeal–bacterial mutualism. Proc Natl Acad Sci.

[CR110] Sauer S, Luge T (2015). Nutriproteomics: facts, concepts, and perspectives. Proteomics.

[CR111] Sims EK (2013). Divergent compensatory responses to high-fat diet between C57BL6/J and C57BLKS/J inbred mouse strains. Am J Physiol-Endocrinol Metab.

[CR112] Sparks LM, Xie H, Koza RA, Mynatt R, Hulver MW, Bray GA, Smith SR (2005). A high-fat diet coordinately downregulates genes required for mitochondrial oxidative phosphorylation in skeletal muscle. Diabetes.

[CR113] Suez J (2014). Artificial sweeteners induce glucose intolerance by altering the gut microbiota. Nature.

[CR114] Sun SY, Ji YW, Kersten S, Qi L (2012). Mechanisms of inflammatory responses in obese adipose tissue. Annu Rev Nutr.

[CR115] Sundsten T, Ortsater H (2009). Proteomics in diabetes research. Mol Cell Endocrinol.

[CR116] Sverdlov AL (2015). High fat, high sucrose diet causes cardiac mitochondrial dysfunction due in part to oxidative post-translational modification of mitochondrial complex II. J Mol Cell Cardiol.

[CR117] Takamura T (2008). Obesity upregulates genes involved in oxidative phosphorylation in livers of diabetic patients. Obesity.

[CR118] Telese F, Gamliel A, Skowronska-Krawczyk D, Garcia-Bassets I, Rosenfeld MG (2013). “Seq-ing” insights into the epigenetics of neuronal gene regulation. Neuron.

[CR119] Temelkova-Kurktschiev T, Stefanov T (2012). Lifestyle and genetics in obesity and type 2 diabetes. Exp Clin Endocrinol Diabetes.

[CR120] Tilgner H, Grubert F, Sharon D, Snyder MP (2014). Defining a personal, allele-specific, and single-molecule long-read transcriptome. Proc Natl Acad Sci USA.

[CR121] van de Bunt M (2013). The miRNA profile of human pancreatic islets and beta-cells and relationship to type 2 diabetes pathogenesis. PLoS ONE.

[CR122] van den Berghe G, Bronfman M, Vanneste R, Hers HG (1977). The mechanism of adenosine triphosphate depletion in the liver after a load of fructose. A kinetic study of liver adenylate deaminase. Biochem J.

[CR123] Via M (2012). The malnutrition of obesity: micronutrient deficiencies that promote diabetes. ISRN Endocrinol.

[CR124] Volkmar M (2012). DNA methylation profiling identifies epigenetic dysregulation in pancreatic islets from type 2 diabetic patients. EMBO J.

[CR125] Vrieze A (2012). Transfer of intestinal microbiota from lean donors increases insulin sensitivity in individuals with metabolic syndrome. Gastroenterology.

[CR126] Walker A (2014). Distinct signatures of host-microbial meta-metabolome and gut microbiome in two C57BL/6 strains under high-fat diet. ISME J.

[CR127] Waller-Evans H (2013). Nutrigenomics of high fat diet induced obesity in mice suggests relationships between susceptibility to fatty liver disease and the proteasome. PLoS ONE.

[CR128] Waller-Evans H (2013). Nutrigenomics of high fat diet induced obesity in mice suggests relationships between susceptibility to fatty liver disease and the proteasome. PLoS ONE.

[CR129] Wang Z, Gerstein M, Snyder M (2009). RNA-Seq: a revolutionary tool for transcriptomics. Nat Rev Genet.

[CR130] Wang TJ (2011). Metabolite profiles and the risk of developing diabetes. Nat Med.

[CR131] Wen HT (2011). Fatty acid-induced NLRP3-ASC inflammasome activation interferes with insulin signaling. Nat Immunol.

[CR132] Wolden-Kirk H (2013). Unraveling the effects of 1,25(OH)(2)D-3 on global gene expression in pancreatic islets. J Steroid Biochem Mol Biol.

[CR133] Wolden-Kirk H (2014). Discovery of molecular pathways mediating 1,25-dihydroxyvitamin D3 protection against cytokine-induced inflammation and damage of human and male mouse islets of Langerhans. Endocrinology.

[CR134] Wu GD (2011). Linking long-term dietary patterns with gut microbial enterotypes. Science.

[CR135] Wu YB (2014). Multilayered genetic and omics dissection of mitochondrial activity in a mouse reference population. Cell.

[CR136] Xia J (2014). Transcriptome analysis on the inflammatory cell infiltration of nonalcoholic steatohepatitis in Bama minipigs induced by a long-term high-fat, high-sucrose diet. PLoS ONE.

[CR137] Zhang LH, Perdomo G, Kim DH, Qu S, Ringquist S, Trucco M, Dong HH (2008). Proteomic analysis of fructose-induced fatty liver in hamsters. Metab-Clin Exp.

[CR138] Zhao YQ, Huang JF (2011). Reconstruction and analysis of human heart-specific metabolic network based on transcriptome and proteome data. Biochem Biophys Res Commun.

